# Effectiveness of psychological interventions for remote workplace trauma: an evidence review

**DOI:** 10.1093/occmed/kqag092

**Published:** 2026-08-13

**Authors:** S K Brooks, R Guruswamy, N Greenberg

**Affiliations:** King’s College London, London, UK; King’s College London, London, UK; King’s College London, London, UK

## Abstract

**Background:**

Workers in specialist and other high-risk roles can experience secondary traumatic stress and related symptoms through routine exposure to distressing material, yet organisational responses vary widely. Evidence for the effectiveness of commonly used approaches (e.g. screening, training, counselling/debriefing, mindfulness/resilience) remains uncertain and fragmented.

**Aims:**

To evaluate the effectiveness of mental health screening, training and psychological interventions in reducing psychological harm for employees exposed to potentially traumatic material.

**Methods:**

Five databases (Web of Science, Medline, PsycInfo, Embase, Scopus) were searched in March 2025, using a rapid review approach. A total of 9581 citations were screened for relevance; 28 studies were included in the final review.

**Results:**

Quantitative findings showed no benefit of mental health screening, routine counselling, monitoring/periodic assessment, mindfulness or resilience programs; mixed effects for training and neutral-to-adverse findings for debriefing. Qualitative findings favoured supervision and access to informal support; debriefing/counselling results were mixed. Cross-sectional studies suggested associations between better organisational support and fewer psychological stress symptoms.

**Conclusions:**

Overall, efficacy evidence is limited and inconsistent across approaches. However, the strongest evidence suggests that organisations should avoid mandating psychological debriefing.

Key learning points **What is already known about this subject:**

- Workers who routinely handle distressing digital/case material face elevated risks of secondary traumatic stress, PTSD-type symptoms and burnout.
- Organisations commonly use screening, training, counselling/check-ins, debriefing and mindfulness/resilience, but single-session debriefing is discouraged, and screening has limited predictive value.
- The evidence base is fragmented and largely observational with heterogeneous interventions and outcomes, justifying a focused synthesis.

**What this study adds:**

- Across 28 studies, there is no demonstrated benefit for psychological screening, routine counselling/check-ins, periodic assessments, mindfulness or resilience programmes.
- Psychological debriefing shows neutral-adverse findings; training effects are inconsistent, and one apparent improvement was attributable to attrition.
- Qualitative accounts value supervision and access to help; cross-sectional analyses associate broader organisational supports with fewer symptoms, though causality cannot be inferred.

**What impact this may have on practice or policy:**

- Avoid mandatory psychological debriefing in services supporting personnel exposed to distressing material.
- Treat other commonly used approaches as unproven pending robust evaluation; redirect resources away from low-value activities.
- Given the limited, heterogenous evidence base, prioritize further development, specification and evaluation of training and system-level organisational interventions.

## Introduction

Frequent occupational exposure to potentially traumatic material can negatively affect the mental health of personnel in high-risk roles (e.g. first responders, social workers) [1]. There is a growing interest in secondary traumatic stress (STS) among such workers, which refers to symptoms caused by indirectly being exposed to trauma affecting other people [2]. STS can impair professionals’ wellbeing, mental health, job performance and overall quality of life. Organisations with staff who are indirectly exposed to trauma commonly use pre-employment screening, training interventions and psychological support programmes to mitigate the risk of STS [1]. However, the effectiveness of these interventions is unclear [3,4].

This rapid evidence review aims to synthesize the current evidence regarding intervention efficacy in reducing STS and associated mental health problems among workers exposed to indirect trauma. In particular the review looked at exposure to distressing content material (DCM) which refers to digital evidence (e.g. pictures, videos) which has been linked to STS symptoms in law-enforcement personnel, including intrusive thoughts, avoidance and changes in arousal and mood [5]. Based on our preliminary scoping, no prior review has addressed DCM in law enforcement, and interventions available to support staff. Given the fast-changing nature of law-enforcement work, we used a rapid evidence review to establish a timely, baseline synthesis of intervention effectiveness to inform organisational policies and expert-witness testimony. Unlike a scoping review, this helped prioritize the evaluation of intervention effects, rather than mapping them. Following Cochrane Rapid Reviews guidance [6], we streamlined methods (pre-specified scope, five databases, English-language limits, single-reviewer screening with team verification, narrative synthesis) appropriate for a heterogeneous evidence base, while transparently reporting our search and selection process, and key PRISMA items, where feasible.

## Methods

This review focused on workers routinely exposed to DCM, such as specialist police units (e.g. cybercrime units, e-crime units, rape crime units, child abuse investigators, body recovery units, family liaison officers and civilian crime scene investigators). These units specialize in dealing with traumatic material not directly threatening their lives or physical health, and experience repeated, indirect exposure to highly distressing material, qualitatively different from acute, frontline incidents. UK policing bodies flag elevated welfare risks and the need for tailored organisational support for these teams [7].

We selected interventions that map onto established workplace mental health frameworks documented by the World Health Organisation [8]. These included: psychological screening of officers prior to commencing work in specialist units; training of officers prior to or during work in specialist units; instruction of officers prior to or during work in specialist units; training of line managers to identify those requiring psychological referral; monitoring of officers; periodic psychological assessments; counselling following exposure to trauma or stress during the course of work; debriefing following exposure to trauma; provisions for confidential self-referral in cases of concern about psychological ill-health following trauma exposure, and any other psychosocial workplace wellbeing interventions.

Our search strategy therefore comprised three search strings: (1) occupational terms (e.g. law enforcement); (2) intervention-related terms (e.g. screening, training) and (3) terms signifying specialist work (e.g. cybercrime, body recovery). The full strategy presented in Table 1 was used to search five electronic databases (Web of Science, Medline, PsycInfo, Embase and Scopus) on 23 March 2025.

**Table 1:** Search strategy

Search String 1: occupational terms		Search String 2: intervention-related terms		Search String 3: terms relating to type of work
police* or ‘law enforcement’ or employee* or staff or worker* or personnel or officer* or ‘content moderator’ or fire? fighter* or ‘fire officer*’ or paramedic* or ‘war correspondent*’ or humanitarian or forensic or ‘high risk work*’ or ‘high risk job*’ or ‘high risk occupation*’ or ‘user generated content’ or media	AND	screening or training or instruction* or monitoring or referral* or counselling or ‘psychological assessment*’ or debrief* or CISD or signpost* or ‘secondary trauma*’ or ‘vicarious trauma*’ or ‘type 2 trauma*’ or psychoeducation* or psycho-education*	AND	‘specialist unit*’ or ‘specialised unit*’ or ‘specialized unit*’ or ‘cyber crime*’ or ‘e-crime*’ or ‘cyber support*’ or ‘cyber investigation*’ or ‘internet protection’ or ‘rape crime*’ or ‘rape victim*’ or ‘child abuse’ or ‘body recovery’ or ‘family liaison’ or ‘crime scene investigat*’

Records retrieved from Web of Science, MEDLINE, PsycInfo, Embase and Scopus on 23 March 2025. Final search combined String 1 **AND** String 2 **AND** String 3.

Studies were included if they: (1) were published in peer-reviewed journals in the English language; (2) contained primary data; (3) recruited individuals regularly exposed to traumatic content as part of their roles; (4) considered psychological wellbeing or mental health-related outcomes and (5) examined the effectiveness (or perceived effectiveness) of organisational interventions to mitigate psychological risk.

Studies were downloaded to EndNote (Version 21) where duplicates were immediately removed. Titles and abstracts were screened by SKB and any clearly irrelevant to the research question were excluded. Full texts of remaining citations were read, and any not meeting the inclusion criteria were excluded. One reviewer (SKB) also completed data extraction and narrative synthesis, a piloted spreadsheet was designed, with headings for: year of publication; country; research design, number of participants, socio-demographic characteristics of participants, occupation(s) of participants, intervention(s) explored, outcomes assessed and key results. Data were analysed by intervention type.

## Results

Database searches yielded 9581 citations, of which 4560 were duplicates and excluded. Title screening led to the exclusion of a further 4302 citations, while abstract screening excluded 662. Full texts of all remaining citations were searched for; three could not be located, 33 failed to meet all inclusion criteria and 21 were ultimately included. Hand searching generated a further seven studies, producing a total of 28 included studies. An overview of the included studies is provided in Appendix 1 (available as Supplementary material at *Occupational Medicine* Online).

The 28 included studies spanned 10 countries (United States (US) [*n* = 13], United Kingdom (UK) [*n* = 4], Netherlands [*n* = 3], Australia and Israel [*n* = 2 each] and Brazil, Canada, Denmark, Norway, Serbia [*n* = 1 each]). Designs were mainly quantitative (*n* = 19**:** 12 cross-sectional, 4 longitudinal and 3 experimental/quasi-experimental including one randomized trial), with 3 mixed-methods and 6 qualitative studies. Sample sizes ranged from 10 to 1963 participants. Occupational groups included police/law-enforcement (*n* = 10), child-welfare/social-service workers (*n* = 8), victim/domestic-violence advocates (*n* = 3), mental health clinicians (*n* = 1), firefighters (*n* = 1), public-safety personnel (*n* = 1), anti–sex-trafficking professionals (*n* = 1) and other/unspecified (*n* = 3). Below, we summarize evidence for all interventions of interest.

Regarding psychological screening prior to commencing work, Powell *et al.*’s police officer participants believed pre-employment selection was important, although majority felt more robust selection processes were needed [7]. However, this qualitative study did not test the actual effectiveness of pre-employment screening. No studies explored the effectiveness of pre-employment psychological screening for the target occupational groups. However, a quantitative study by Kunst *et al.*, found that formal or informal selection of staff as police family liaison officers was not significantly associated with either STS or post-traumatic growth [9].

Five studies examined training prior to commencing work, or during work. Mitchell *et al.* found that preparing forensic examiners/investigators for exposure to child sexual abuse material (CSAM) was significantly related to fewer mental health symptoms and higher wellbeing, while attending a training programme on CSAM was significantly related to higher wellbeing [10]. Damasio *et al.* found that a training programme which instructed professionals to use cognitive behavioural therapy for working with young victims of sexual abuse, stabilized emotional exhaustion levels, while depersonalisation decreased; in comparison, controls experienced increased emotional exhaustion and depersonalisation, as well as reduced professional efficacy [11]. However, Benuto *et al.* found that attending training, conferences and workshops was not significantly associated with STS in victim advocates [12]. Nisbet *et al.* [13] evaluated the effect of Emotional Resilience Skills Training (a 13-week mental health programme focusing on aversive reactions to emotions which may result in avoidance) on suicidal ideation in public-safety personnel. Although initial results indicated reduced suicidal ideation at the 1-year follow-up, this was found to be due to study attrition. Additionally, Turgoose *et al.* [14] carried out a training intervention designed to increase knowledge of compassion fatigue, STS and burnout in police working with rape victims. Qualitatively, participants expressed relief at being able to name their own feelings; however, the study did not formally evaluate whether participants experienced improvements in mental health or wellbeing.

We found no studies exploring the effectiveness of instruction, prior to, or during work, for target occupational groups. Similarly, we found no studies exploring the effectiveness of training line managers in identifying staff in need of psychological referral, or any studies exploring the effectiveness of making provision for confidential self-referral in cases of concern following exposure to trauma.

Regarding monitoring of staff and periodic psychological assessment, participants in a qualitative study of police officers investigating internet child exploitation reported peer monitoring to be tokenistic and inadequately implemented [7]. Mitchell *et al.* found that participants frequently requested mandatory mental health check-ins to protect their mental health, but did not explore the effectiveness of periodic assessments [15]. One study of family liaison officers found that ‘mental check-ups’ were not associated with either STS or post-traumatic growth [9]. However, the study did not specify whether checkups were mandatory or, if not, what the uptake was.

Regarding counselling following trauma or stress exposure, a qualitative police officer study did not find psychologist appointments helpful, for various reasons (e.g. perceiving psychologists as under-qualified; believing psychologists were uncomfortable hearing case-related material) [7]. Conversely, Mitchell et al interviewed child sexual abuse investigators who considered that therapy and mental health services, including counselling, peer support and venting without fears of stigma were important [15], but there was no quantitative assessment of these interventions. Only one study examined the effectiveness of counselling: Benuto *et al.* found that support groups and counselling did not significantly affect STS in victim advocates [12].

Regarding debriefing following exposure to trauma, one qualitative study found that participants felt negatively about formal debriefing, describing it as ‘tokenistic’ and ineffective [7]. Two qualitative studies revealed that participants felt positively about debriefing: Russ *et al.*’s participants believed debriefing increased resilience, and all appeared to value the opportunity for reflection with supervisors and peers [16]. Loncar *et al.*’s participants reported wanting a management initiative on debriefing to address mental health stigma, organized follow-ups and a formal, structured debriefing system as a preventive measure [17]. Only three studies quantitatively evaluated the effectiveness of debriefing in protecting mental health or psychological wellbeing: Harris *et al.* [18] found critical incident stress debriefing had a weak inverse correlation with negative affectivity, weak positive correlation with positive world assumptions, but no relationship with PTSD. Carlier *et al.* found that, among police who responded to a civilian plane crash, there was no difference between debriefed and non-debriefed officers after eight months, but after eighteen months those who had undergone debriefing exhibited significantly more disaster-related hyperarousal symptoms [19]. Additionally, 1 week post-trauma, debriefed police officers exhibited significantly more post-traumatic stress symptoms than non-debriefed officers, although there were no significant differences at 6-month follow-up [20].

In relation to mindfulness, one study by Benuto *et al.* found that mindfulness-based interventions did not significantly affect STS in victim advocates [12].

With respect to other psychological interventions, majority of included studies considered the effectiveness of workplace psychological interventions other than screening and training: namely, mindfulness, supervision, organisational support, organisational activities and resilience interventions.

Four qualitative studies suggested participants perceived supervision to be important. Foley *et al.*’s qualitative study revealed that good supervision was perceived by police officers to be an important protective factor against poor wellbeing [21]. Similarly, Russ *et al.*’s child protection worker participants believed supportive supervision was important, and created a sense of safety in emotional processing, learning and decision-making [16]. Wymer *et al.* found that mental health clinicians working with child sexual abuse survivors believed that supervision sessions, relationships with supervisors and supervisor traits (e.g. non-judgmental, trustworthy, proactive in terms of checking in with employees) influenced their management of work-related challenges [22]. Denk-Florea *et al.*’s study of law enforcement personnel explored how participants perceived workplace support to affect them [5]. Participants believed that managerial support aided their wellbeing: in particular, managers who promoted a relaxed work environment, were approachable, understood the impact and nature of the job and provided reassurance when needed. Managers lacking in these skills appeared to exacerbate distress: for example, one participant described developing anxiety and depression, after having to continuously prove their distress and unsuccessfully seek support from management.

However, most quantitative studies failed to support the idea of supervision protecting wellbeing. One study showed that employees who reported receiving more support from their supervisors reported significantly less burnout [23]. Another found that supervisor support was significantly associated with lower STS in child exploitation personnel in the US, but not in the UK [24]. One study found evidence that supervision on the personal impact of traumatic work was associated with significantly higher secondary traumatisation, disengagement and emotional exhaustion, suggesting that supervision may actually increase perceived distress [25]. In the same study, case supervision moderated the relationship between exposure to child abuse cases and both STS and burnout, suggesting that while case supervision was not directly protective against work-related distress, it could moderate the effect of exposure. However, majority of studies which carried out multivariate regressions found no significant associations between supervisory support and STS [26–30], post-traumatic growth [30], or vicarious trauma [26].

Regarding organisational support, Rienks’ correlational analysis showed a meaningful association between STS and ‘organisational support’ [31], while North *et al.* found that employees who reported receiving more organisational support also reported significantly less burnout [23]; however, it is unclear exactly what ‘organisational support’ entailed in these studies. Choi found that workers with ‘sociopolitical support’ from their superiors and colleagues, and with greater access to ‘strategic information’, experienced significantly lower levels of STS [27]; however, again, these constructs were not clearly defined.

In relation to organisational activities, Whitt-Woosley and Sprang [32] and Sprang *et al.* [33] found both STS and burnout were significantly associated with organisational activities relating to safety, resilience, policies, leader practices and organisational practices. Activities associated with resilience and safety were most strongly associated with STS, and activities associated with leadership and policies were most strongly associated with burnout. However, it is unclear from the studies what exactly these activities entailed. Mitchell *et al.* examined mental health symptoms in forensic investigators exposed to child sexual abuse material, and found those working in agencies with Officer Wellness Programmes reported significantly lower mental health symptoms, lower post-traumatic stress symptoms and higher wellbeing [10]. Specific policies and practices associated with mental health included ample time off, daily opportunities to debrief and providing regular administrative updates about positive outcomes from child sexual abuse cases.

Regarding resilience interventions, Orsi-Hunt *et al.* [34] explored the effectiveness of a 24-week programme (the ‘Resilience Alliance’ intervention) designed to mitigate the impact of STS. This intervention’s objectives included recognising work-related stressors and responses; using cognitive behavioural therapy strategies to reframe responses; understanding avoidance management techniques, control, self-defeating statements and encourage hopefulness; enhancing self-regulation abilities and demonstrating the connection between social support and emotional wellbeing. Participation in this intervention did not reduce STS or burnout, or increase in resilience.

## Discussion

We found no robust evidence showing beneficial impacts of screening, instruction, line managers training to identify staff needing psychological referral, or provisions for confidential self-referrals in protecting the mental health of workers dealing with DCM. We note that a previous review considering the effectiveness of screening in a disaster relief workers found little evidence that pre-employment and pre-deployment screening could indicate resilience [3]. Likewise, broader law enforcement literature suggests screening instruments add minimal, if any, value to the selection process [35,36]. Taken together, these findings suggest that difficulties in prospectively identifying psychological vulnerability is not unique to specialist police populations but represents a broader challenge across trauma-exposed occupations [37].

The reviewed studies showed no significant improvements in psychological wellbeing or mental health following monitoring or periodic psychological assessments, counselling, debriefing, mindfulness interventions or a resilience-focused intervention; notably, psychological debriefing/routine trauma counselling was associated with poorer outcomes in two studies. Although qualitative data suggested participants valued opportunities to discuss their mental health, quantitative data failed to show significant associations with improved mental health or wellbeing. This may indicate that interventions viewed positively by employees do not necessarily produce detectable effects. Our findings are consistent with previous syntheses showing that single-session debriefing is ineffective and potentially harmful [38], and align with 2018 NICE guidelines advising against routine psychological debriefing after trauma [39].

Evidence regarding training was mixed, with two studies suggesting training was protective of mental health [10,11], while others found no association. Mixed evidence was also found for the effectiveness of supervision; most studies suggested supervision was not associated with mental health or wellbeing, but three found significant associations [23–25]. This variability may reflect differences in how training and supervision are delivered and implemented, rather than demonstrating an absence of effect. Recent workplace mental health guidance indicates there may still be value in equipping managers with skills to recognize and respond appropriately to mental health concerns and fostering psychologically supportive supervisory relationships [8,40].

A small number of studies suggested ‘organisational support’, ‘organisational activities’ (associated with resilience, safety, leadership, policies) and Officer Wellness Programmes may be protective of mental health [10,23,31–33]. However, these concepts were poorly defined, making it difficult to identify which specific organisational practices may be beneficial. Nevertheless, these findings tentatively suggest that broader organisational culture and supportive working environments may be more important than individual-level interventions alone; more rigorous research is needed to better understand the benefits.

Both included studies and our review have limitations that warrant consideration. Most constituent studies used cross-sectional or qualitative designs with self-selected samples, relied on self-report outcomes and showed high heterogeneity in occupational groups, intervention types, study designs, exposure profiles and measures of STS/burnout. Sample sizes also varied substantially. Few trials included controls or long-term follow-up; ‘organisational support/activities’ were often poorly specified, and most studies were US-based. Collectively, this constrains causal inference, comparability and generalisability; findings should therefore be interpreted cautiously. We opted to capture a range of occupations exposed to potentially traumatic material, in both digital and in-person contexts; however, we acknowledge this introduced further heterogeneity and future reviews may benefit from distinguishing workers primarily exposed to digital content from those exposed through direct interpersonal contact, as mechanisms of occupational stress may differ. The review was conducted as a rapid review with streamlined methods. Searches were run in a finite set of databases, on a single date, and restricted to English-language records; grey literature was not systematically searched. Wider searches, using additional databases, grey literature and larger search strategies may have located additional studies. Furthermore, data screening (titles, abstracts and full texts), selection and extraction, was conducted by one author, without independent check of eligibility decisions or extracted data. These choices may increase the risk of study-selection, extraction and classification error and may overlook relevant but non-indexed evidence, biasing findings towards published positive results.

In conclusion, high-quality evidence to guide organisations seeking to protect the mental health of workers exposed to traumatic content remains limited. Existing evidence does not support routine screening, monitoring, debriefing or referral-based approaches as standalone preventive strategies. More promising avenues may lie in strengthening organisational support and promoting psychologically informed supervision; however, these approaches require more rigorous evaluation. Current evidence is insufficient to support firm recommendations regarding how organisations can protect the wellbeing of staff indirectly exposed to trauma and further high-quality studies with clearly defined interventions are needed.

## Supplementary Material

kqag092_Supplementary_Data

## Data Availability

Data underlying this article are available in the article and in its Supplementary Material. EQUATER Network checklist utilized (only if applicable): Not applicable (rapid review conducted).
