# Supplementary material for: Effectiveness of psychological interventions for remote workplace trauma: an evidence review
Source: Occup Med (Lond). 2026 Aug 13;76(6):417–23. doi: 10.1093/occmed/kqag092 (PMC13673501; doi:10.1093/occmed/kqag092)
Supplement: Supplementary file 1 [file kqag092_supplementary_data.docx]

**Appendix 1. Characteristics of reviewed studies**

| **Authors (year)** | **Country** | **Study design** | **Occupational group(s)** | **Number of participants** | **Socio-demographic characteristics** | **Intervention(s) / organisational factors** | **Outcomes assessed** |
| --- | --- | --- | --- | --- | --- | --- | --- |
| Benuto et al. (2019) | United States of America | Cross-sectional survey | Victim advocates | 142 | 134 female, 8 male; age range 21-72 years | Organisational support, including training / conferences / workshops; mindfulness-based interventions; support groups / counselling | Secondary traumatic stress |
| Bolić (2019) | Serbia | Cross-sectional survey | Child welfare professionals | 135 | 85.9% female; age group most represented was 35 and under (34.1%) | Provision of regular and group supervision | Vicarious trauma; compassion satisfaction; compassion fatigue |
| Bourke & Craun (2014) | United States of America; United Kingdom | Cross-sectional survey | Child exploitation personnel | 965 (677 in USA; 288 in UK) | Not reported | Support from coworkers and supervisors (e.g. ‘how easy is it to talk to your immediate supervisor?’) | Secondary traumatic stress |
| Carlier et al. (1998) | Netherlands | Longitudinal | Police officers who had responded to a civilian plane crash | 49 debriefed, 59 non-debriefed | 95 male, 10 female; mean age 37 years | Debriefing | Post-traumatic stress symptomatology |
| Carlier et al. (2000) | Netherlands | Pre-test post-test control group design | Police officers exposed to trauma (e.g. accidents involving death or serious injury; failed resuscitations) | 243 | External control group (officers who had undergone traumas before occupational debriefing was introduced in 1992, n=75): 80% male, 20% female; mean age 32.4 years  Experimental group (traumatised officers who received debriefing, n=86): 70% male, 30% female; mean age 28.9 years  Internal control group (officers who declined the offer of debriefing, n=82): 65% male, 35% female; mean age 31.7 years | Debriefing (three debriefings, approximately 24hours, 1 month and 3 months after the incident) | State-trait anxiety; post-traumatic stress disorder; dissociative experiences |
| Choi (2011) | United States of America | Cross-sectional survey | Social workers assisting family violence or sexual assault survivors | 154 | 78.6% female; age not reported | Organisational support (sociopolitical support, access to strategic information, access to resources, and organisational culture); quality of supervision | Secondary traumatic stress |
| Damasio et al. (2014) | Brazil | Quasi-experimental design with pre-post tests in experimental and comparison groups | Psychologists involved in working with victims of child and adolescent sexual abuse | 30 (experimental group n=19, comparison group n=11) | Experimental group: 84.5% female, mean age 36.9 years  Comparison group: 100% female, mean ae 35 years | Training programme designed to instruct professionals in the use of evidence-based cognitive behavioural therapy for working with child and adolescent victims | Extent to which training indirectly benefited professionals by reducing job stressors and enhancing job skills, and reducing burnout and job strain |
| Denk-Florea et al. (2020) | United Kingdom | Semi-structured interviews | Law enforcement personnel  (59% digital forensics technicians, 18% managers, 13% investigators, 5% supervisor, 5% detective) | 22 | 16 male; mean age 40.65 years | Participants asked whether they felt supervision and psychological support affected their welfare | Perceived personal welfare |
| Foley et al. (2024) | United Kingdom | Qualitative interviews | Police officers who investigate rape and child abuse | 23 | 63% female, 37% male; 21% aged 30-35, 16% aged 36-40, 31.5% aged 41-45, 31.5% aged 45+ | Organisational support | Interviews assessed coping |
| Harris et al. (2002) | United States of America | Cross-sectional survey | Firefighters | 660 | 97.3% male, 2.6% female (1 participant did not report gender); modal age group 30-39 years | Critical incident stress debriefing | Avoidance coping; negative affectivity; world assumptions; post-traumatic stress disorder symptoms |
| Kunst et al. (2017) | Netherlands | Mixed-methods: Qualitative interviews and cross-sectional surveys | Police family liaison officers | 224 | 56.3% male; mean age 47.6 years | Having been (in-) formally selected for working as a PFLO; availability of a mental checkup in the region of employment; supervisor and co-worker support | Secondary posttraumatic growth; secondary traumatic stress |
| Loncar et al. (2025) | Norway | Qualitative interviews | Police employees investigating child abuse | 16 | 50% female, 50% male; age not reported | Organisational resources | Interviews assessed challenges and what were perceived to be the most important resources for coping |
| Mitchell et al. (2022) | United States of America | Mixed-methods: Qualitative interviews and cross-sectional surveys | Internet Crimes Against Children Task Force Network | Interviews: 54  Surveys: 155 | 19% female; age not reported | Preparation for first exposure; existence of Officer Wellness Program; other options for staff that could help promote wellness | Participants were asked about resources that could promote wellness |
| Mitchell et al. (2023) | United States of America | Cross-sectional survey | Forensic examiners / investigators exposed to child sexual abuse material | 500 | 61.4% male, 37.4% female, 0.2% non-binary, 1% declined to answer; age group most represented was 35-44 (39.8%) | Officer Wellness Program; other specialised training around wellness; other services available for wellbeing e.g. peer counsellors | Depression; anxiety; posttraumatic stress symptoms; subjective wellbeing |
| Nisbet et al. (2025) | Canada | Longitudinal prospective sequential experimental cohort design | Public safety personnel | 186 | 60.5% male; mean age not reported, age group most reported was 30-39 years (48.4%) | Emotional Resilience Skills Training – a 13-week mental health training programme focusing on aversive reactions to emotions which may result in emotional avoidance | Suicidal ideation, planning and attempts |
| North et al. (2025) | United States of America | Longitudinal mixed-methods | Victim advocates working within children’s advocacy centers | 190 | 93% female; majority aged between 20-34 years (56.1%) | Perceived organisational support; perceived supervisory support | Burnout; turnover intentions |
| Orsi-Hunt et al. (2023) | United States of America | Cluster randomised controlled trial | Child welfare professionals | 192 (treatment condition) vs 183 (no intervention) | 87% female, 13% male; mean age 34.7 years | ‘Resilience Alliance’ model; a 24-week programme designed to mitigate the impact of secondary traumatic stress; objectives include recognising work-related stressors and responses; using cognitive behavioural therapy strategies to reframe responses; understanding techniques to manage avoidance, control, self-defeating statements, and encourage hopefulness; enhance self-regulation abilities; and demonstrate the connection between social support and emotional wellbeing. 24 sessions are held weekly for six months. Participants also received weekly text messages reminding them of key skills and messages. For an additional 6 months after the intervention, participants had a monthly Peer Support Group | Secondary traumatic stress; work-related burnout; resilience; turnover intentions; actual turnover assessed from Human Resources data |
| Powell et al. (2014) | Australia | Qualitative interviews | Police officers investigating internet child exploitation | 32 | 22 male, 10 female; age not reported | Organisational strategies facilitating or inhibiting ability to perform investigations | Interviews assessed coping strategies and perceived impact of organisational strategies |
| Rienks (2020) | United States of America | Longitudinal | Child welfare caseworkers | 1,963 (baseline); 653 (3-year follow-up) | 86.5% female; age not reported | Organisational support (measured by 4 items e.g. ‘My agency really cares about my wellbeing’) | Burnout; coping; secondary traumatic stress |
| Russ et al. (2020) | Australia | Qualitative interviews | Child protection workers | 24 | 18 female, 6 male; age not reported | Organisational influences on resilience | Perceived resilience |
| Skar et al. (2023) | Denmark | Cross-sectional survey | Child protection workers | 667 | Not reported | Receipt of supervision at work on their casework and/or on the personal impact of their work | Secondary traumatisation; burnout |
| Slattery & Goodman (2009) | United States of America | Cross-sectional survey | Domestic violence advocates | 148 | 100% female; mean age 36 years | Quality of supervision | Secondary traumatic stress |
| Sprang et al. (2023) | United States of America | Cross-sectional survey | Professionals working with survivors of sex trafficking | 583 | 80.6% female, 1.9% non-binary or other, 17.5% male; mean age not reported, age group most represented was 35-44 years (29.6%) | The extent to which participants perceived organisation as STS-informed and responsive to the impact of STS | Secondary traumatic stress |
| Turgoose et al. (2017) | United Kingdom | Follow-up study (assessment 8-10 weeks after training) | Police officers working with rape victims | 142 at baseline (n=38 completed follow-up measures) | 62% female; age not reported | 90-minute training session which included psychological education (about stress, compassion fatigue, secondary traumatic stress and burnout) and self-help strategies (including identifying stress, self-care, stress management, social support mindfulness, compassion satisfaction( | Compassion fatigue, compassion satisfaction and burnout; secondary traumatic stress; knowledge of the concepts covered in the training |
| Weiss-Dagan et al. (2016) | Israel | Cross-sectional survey | Social service workers and child protection workers | 255 | 94.2% female, 5.8% male; mean age 41.6 years | Supervision (participants rated the effectiveness of supervision with a 7-item scale e.g. ‘rate the extent of your satisfaction with supervision’) | Secondary traumatic stress |
| Weiss-Dagan et al. (2020) | Israel | Cross-sectional survey | Social workers who have worked with abused children | 255 | 94.2% female, 5.8% male; mean age 41.6 years | Supervision (participants rated the effectiveness of supervision with a 7-item scale e.g. ‘rate the extent of your satisfaction with supervision’) | Post-traumatic growth; secondary traumatic stress; role stress |
| Whitt-Woosley & Sprang (2023) | United States of America | Cross-sectional survey | Child welfare professionals within social service systems | 382 | 87.3% female; mean age 42.52 years | Organisational approaches to secondary traumatic stress prevention and intervention, including activities associated with resilience, safety, policies, leader practices and organisational practices / monitoring | Secondary traumatic stress; burnout |
| Wymer et al. (2020) | United States of America | Qualitative; semi-structured interviews | Mental health clinicians working with child survivors of sexual abuse | 10 | 100% female; age range 25-34 | Provision of supervision | Experiences with secondary traumatic stress symptoms and perceptions of beneficial supervision practices |
